# A family case of early onset juvenile idiopathic arthritis with uveitis: lessons of the past and the present

**DOI:** 10.1186/1546-0096-12-S1-P208

**Published:** 2014-09-17

**Authors:** Irina Nikishina, Svetlana Arsenyeva, Dmitry Alekseev

**Affiliations:** 1 Pediatric, Nasonova research Institute of Rheumatology, Moscow, Russian Federation

## Introduction

Early onset of juvenile idiopathic arthritis (eoJIA) associated with pauci?polyarthicular joints involvement, antinuclear factor and uveitis is a special subtype of JIA. Generally accepted opinion of pediatric rheumatologists this JIA category isn`t belong to ankylosing spondylitis (AS)-related group of diseases.

## Objectives

To present clinical observation of a family case (mother and daughter) of eoJIA with uveitis, homogenous clinical picture in the disease beginning and manifestation of severe AS in the mother in adulthood.

## Methods

Cases report.

## Results

Female patient was born in 1978. Disease onset was reported when she was 3 years old from arthritis of right knee and developed polyarthritis within 2 years. By the 3^rd^ year of disease duration bilateral uveitis involved. She was rtreated by NSAIDs treatment, intra-articular glucocorticoid (GC) injections, and different (>5) DMARDs consequently; multiple courses of GC pulse therapy, local ocular therapy and repeated surgical treatment of ocular complications, since 1996 - prednisolone (10 mg/day) continuously. Despite the treatment, polyarthritis and hight activity persisted, corneal spot developed. Significant aggravation was reported after the first labors (age 22 years); inflammatory back pain appeared. Ankylosis of neck and sacroiliac joints, osteonecrosis of left femoral head were observed. ive. AS (HLA-B27-negative) was diagnosed after 20 years of disease duration. In 2004 hip replacement of left femoral joint performed. In 2009 after 2^nd^ labors disease progression with high activity and osteonecrosis of right femoral joint developed, that required hip replacement. High activity persisted within next 4 years until adalimumab (ADA) therapy was started. Her daughter, which was born in 2009, developed oligoarthritis in the age of 2. Despite of therapy with NSAIDs, SSZ, i.a. GC injections arthritis was extended to polyarthritis with dactylitis of 3^rd^ fingers of hands and flexor contracture of knee joints. Bilateral uveitis was diagnosed and local eye treatment was initiated. MTX (s/c 12 mg/m2/week) was added to SSZ treatment. 4 months later cyclosporine A prescribed instead of SSZ, repeated i.a.GC injections were needed. ESR was 30 mm/h, C-reactive protein - 52.3 mg/l, HLA-B27 – negative, ANF positive (1/320). Hypertrophy of metaphyses on separate phalanxes by X-ray, bone marrow edema and synovitis of some hand`s fingers by MRA observed. In 2013 ADA was not approved for children younger 4^th^ in Russia. Considering the progression of clinical and functional disorders, local growth distubanceas, periostitis, persistence of active uveitis, and bad outcome of the similar disease in her mother ADA 30 mg s/c once in 2 weeks was added to MTX. Initial effect was good for the arthritis and uveitis; but later, within the next 2 months the efficacy level has somewhat decreased. ADA was increased up to 40 mg per injection with achievement of clinical remission by 1 year of therapy without any adverse event. ADA therapy started in her mother (1^st^ pt) in the same time with good respond.

## Conclusion

This clinical case demonstrates the same type disease in mother and daughter. It illustrates the possibility of AS development in female patients with eoJIA with ANA-associated severe uveitis, despite of HLA B27 negativity. Aggressive treatment strategy, including ADA in a very young patient, dose escalation ADA, seems to be justified in the case of high risk of unfavorable prognosis in order to prevent joint destruction and irreversible uveitis complications.

## Disclosure of interest

None declared.

